# Fragmented Care, Fragmented Safety: Care Coordination and Deprescribing Strategies to Reduce Polypharmacy-Related Harm in Assisted Living—A Public Health Perspective

**DOI:** 10.3390/geriatrics11040109

**Published:** 2026-08-18

**Authors:** Amani Mohammed, Mahmoud Abuhasanein, Martin Wawruch, Viktor Foltin

**Affiliations:** Department of Public Health, St. Elizabeth University of Health and Social Work, 81006 Bratislava, Slovakia; mahmoud.abuhasanein@gmx.de (M.A.); wawruch@vssvalzbety.sk (M.W.); vfoltin@vssvalzbety.sk (V.F.)

**Keywords:** assisted living, polypharmacy, deprescribing, medication safety, care coordination, public health

## Abstract

**Background:** Assisted living houses an older, frailer and more heavily medicated population than ever, yet is often regulated as a social and not a clinical setting with no on-site nursing requirement. Polypharmacy accumulates where medication oversight is thinnest. **Objective:** To map the extent, nature and distribution of research on medication safety in assisted living in the narrow sense, and where the evidence clusters and thins. **Methods:** In this scoping review, PubMed and Web of Science were searched (26 May 2026; window 2010–2025) and records screened against a strict two-gate rule: assisted living in the narrow sense, explicitly labelled and central, plus a central medication-safety or coordination concept. Residential-care and long-term-care populations fell outside it. Titles, abstracts and full texts were screened and charted thematically. **Results:** Twenty-two sources met the strict definition, drawn from four countries: the United States (13), Finland (6), Canada (2) and the United Kingdom (1). Five themes emerged, covering burden (4), safety (4), interventions (4), antimicrobial stewardship (4) and coordination and regulation (6). A further 11 comparison-context reports were kept separately. Every randomised trial was Finnish and came from one research programme. **Conclusions:** Assisted living has become a high-acuity medication environment without matching oversight. The setting-specific literature is real but sparse, and rigorous deprescribing and coordination trials remain scarce. The binding constraint is structural rather than pharmacological, a missing point of clinical accountability that is patterned by regulation, so closing the gap is as much a public-health task as a clinical one.

## 1. Introduction

Assisted living was never meant to be a medical setting. It grew out of a simple and appealing idea, namely that older people who need help with daily life should not have to trade their autonomy for a hospital-like institution, and on those terms it has succeeded, becoming a widely used form of supportive residential housing for older adults. Assisted living is chiefly a North American category of housing with services, most developed and most widespread in the United States [1], with broadly analogous models provided under other names in other high-income countries. In its typical form it offers help with activities of daily living such as bathing, dressing and, in most models, assistance with medications, but without the on-site skilled-nursing presence that defines a nursing home. The residents who live there today, however, are not the residents the model was originally built for. They are older, they carry more chronic conditions, and a large share live with cognitive impairment. That clinical complexity brings medication with it: multiple prescribers, long medication lists and the steady, often invisible accumulation of drugs that no one is clearly responsible for reviewing [2,3].

This is where the setting becomes genuinely difficult from a safety standpoint. Nursing homes, by contrast, are subject to comparatively stronger and more standardised medication-oversight requirements, often including on-site nursing coverage and periodic medication review, and it is against this better-regulated setting that the assisted-living regulation and typology studies in this review define their comparison [1,4]. Assisted living, by contrast, is typically regulated at the state or regional level (most visibly in the United States, though comparable subnational variation exists in other high-income countries), and the rules vary enormously from one jurisdiction to the next [1,5]. Many do not require a registered nurse on site at all. Medications are frequently administered by unlicensed staff working from written instructions [6], and the physicians who prescribe them are often off-site and only loosely connected to the day-to-day picture [7]. The result is a care environment that has taken on the pharmacological profile of a nursing home while retaining the staffing and regulatory profile of housing. That gap between clinical need and oversight capacity is the central public-health concern this review sets out to examine.

The specific medication problems are well recognised in geriatric medicine. Polypharmacy raises the risk of drug–drug interactions, falls and hospitalisation [2,8,9]. Potentially inappropriate medications, especially anticholinergics and psychotropics, are disproportionately harmful in frail older people [10,11,12]. Antibiotics are frequently prescribed on thin diagnostic grounds, feeding resistance and adverse events alike [13,14]. Deprescribing, meaning the planned and supervised withdrawal of drugs that are no longer helping [15,16], is an obvious counter-measure, but it depends on exactly the kind of clinical review and coordination that assisted living is least equipped to provide. Care coordination therefore sits at the heart of the problem: it is both the mechanism through which harm accumulates and the lever through which it might be reduced.

Despite a large general literature on medication safety in older adults, it is unclear how much of that evidence actually speaks to assisted living as a distinct setting rather than folding it into “long-term care” or “care homes” more broadly [4]. Previous systematic reviews have synthesised deprescribing and prescribing-optimisation interventions in older people and in care homes [17,18], but they pool these settings and do not isolate assisted living, whose staffing and regulatory model differs markedly; to our knowledge no previous review has mapped the medication-safety evidence specifically for assisted living, which is the gap this review addresses. A scoping review is well suited to this uncertainty: it maps the size, shape and gaps of a body of evidence rather than pooling it to answer a narrow effect question. We deliberately adopted a strict definition of the setting, assisted living in the narrow sense, so that the map would reflect the setting itself and not the much larger residential-care literature that surrounds it.

Accordingly, this review addresses three questions:

(1) How is research on polypharmacy, deprescribing, potentially inappropriate prescribing, antimicrobial stewardship and care coordination distributed specifically within assisted living, by volume, geography and study design?
(2) What do these studies report about the burden of medication-related harm and about the interventions and coordination structures intended to reduce it?
(3) Where does the evidence cluster, and where does it fall silent, when the setting is held strictly to assisted living rather than residential care in general?

Read together, the studies assembled here raise a single organising question that this review sets out to examine: whether medication-related harm in assisted living is driven less by which drugs are prescribed than by the absence of a defined structure of clinical accountability for reviewing them. The question is whether the core problem is that no single clinician or role is clearly and consistently responsible for periodically reviewing each resident’s entire medication regimen. If so, it would be a gap patterned by regulation rather than by clinical need, one that the current sparse and largely observational evidence can describe but cannot yet close. The five themes that follow are examined not in isolation but for how each one bears on this question, and the Discussion returns to it once the evidence has been mapped.

## 2. Materials and Methods

### 2.1. Protocol and Guidance

The review was conducted and reported in line with the PRISMA extension for scoping reviews (PRISMA-ScR) [19] and drew on the JBI methodology and the Arksey and O’Malley framework as later refined by Levac and colleagues. The eligibility rules, the search approach and the charting fields were defined in writing before screening began, and those provisions, including the handling of opinion pieces without primary data, were applied consistently throughout. The review protocol is registered in the Open Science Framework (OSF; https://osf.io/6m8vt, accessed on 15 July 2026). Registration was retrospective: scoping reviews are not eligible for PROSPERO and the protocol was deposited in OSF after data charting had begun. No protocol amendments were made on the basis of the findings, and this timing is disclosed in the interest of transparency. A completed PRISMA-ScR checklist is provided in Appendix A.

### 2.2. Eligibility Criteria (PCC)

Eligibility followed the population–concept–context (PCC) structure recommended for scoping reviews. Population: older adults residing in assisted living. Concept: medication safety in the broad sense, comprising polypharmacy, deprescribing, potentially inappropriate medications, antimicrobial prescribing and stewardship, and the care-coordination processes surrounding prescribing and medication management. Context: assisted living in the narrow sense. Studies were eligible if they satisfied all three PCC elements and reported primary or protocol-level data. Eligible research types included randomised controlled trials, cohort, cross-sectional and pre–post studies, qualitative and mixed-methods studies, feasibility and pilot studies, systematic reviews and study protocols; opinion pieces, editorials and commentaries without primary data were excluded. Eligibility was further restricted to peer-reviewed reports published in English between 2010 and 2025, with the supplementary-source exception noted in Section 2.3.

Two gates had to be cleared for inclusion. The setting gate required that the study be set explicitly in assisted living and that this setting be central rather than incidental (that is, the study population and analysis had to be located in assisted living, not merely mention it in passing or fold it into a larger, unreported subgroup). Internationally labelled “residential care homes”, residential aged-care facilities, care homes for the elderly and long-term-care or nursing-home populations were treated as outside the narrow definition unless an assisted-living stratum was clearly identifiable and separately reported. The concept gate required that medication safety or care coordination be a central object of the study rather than a background covariate. Studies in which medication appeared only as one adjustment variable, or in which the setting was a nursing home, hospital or emergency department, were excluded. In keeping with the protocol, opinion pieces and commentaries without primary data were also excluded.

This strict framing was a deliberate methodological choice. A large share of the wider literature situated in “care homes” or “long-term care” would qualify under a looser reading of the setting and including it would have produced a map of residential care in general rather than of assisted living in particular. We accepted a smaller, more sharply defined evidence base in exchange for a map that genuinely reflects the setting of interest. Reports that were clearly about medication safety but set in residential care, nursing homes or long-term care were not discarded; they were retained separately as comparison context (see Section 3.5) and were never counted among the assisted-living includes. Here residential care denotes long-term-care settings (care homes, residential aged-care facilities and nursing homes) in which medication oversight is typically more formalised than in assisted living in the narrow sense, through arrangements such as on-site or regularly visiting nursing and mandated medication review whose mix varies by setting and country. These comparison-context sources informed the interpretation only as a contextual backdrop; they were not charted into the thematic synthesis and contributed to no finding about assisted living itself.

### 2.3. Information Sources and Search

Two bibliographic databases were searched, PubMed (MEDLINE) and the Web of Science Core Collection, on 26 May 2026, with the publication window restricted to 2010–2025. The window reflects the fact that dense evidence on deprescribing and polypharmacy accumulated only after the relevant MeSH terms were introduced (2018–2019), with a relevant volume of literature from about 2010 onward. In addition to the database searches, a curated reference collection assembled during protocol development and targeted hand-searching of reference lists were used to identify further eligible reports; because these supplementary sources were not bound by the database date filter, they could post-date the 2010–2025 window, which is why one eligible study published in 2026 (the Shed-MEDS deprescribing pilot) is included.

The search combined setting terms for assisted living and residential care with concept terms for polypharmacy, deprescribing, potentially inappropriate medications, medication safety, antimicrobial prescribing and care coordination. The setting block was deliberately kept broad, taking in residential-care and care-home terms alongside assisted living, so as to maximise sensitivity at the search stage. The narrow assisted-living definition was then applied at screening rather than in the search string itself, which is why the funnel narrows so sharply between the records retrieved and the sources finally included.

The two full search strings, exactly as executed in PubMed/MEDLINE and the Web of Science Core Collection, are provided in the Supplementary Material (Table S1).

PubMed and the Web of Science Core Collection were selected as the two principal databases for biomedical and multi-disciplinary coverage, and the reference lists of included reports were additionally hand-searched. Reliance on two major databases, rather than a wider multi-database search, is acknowledged as a limitation in Section 4.4.

### 2.4. Selection of Sources of Evidence

**Figure 1:**
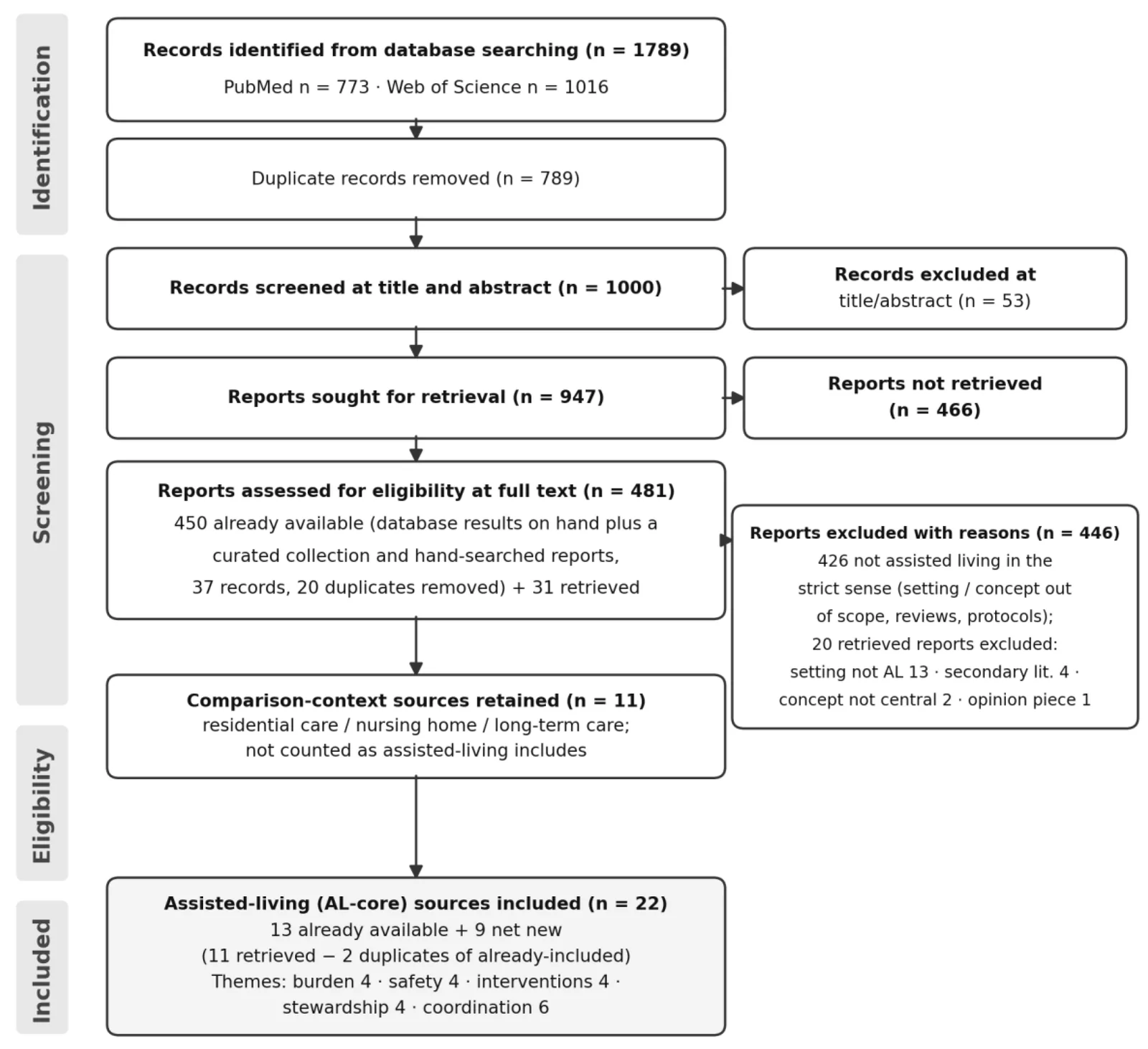
PRISMA-ScR flow of records through the strict assisted-living scoping review.

Screening proceeded in two stages. At the first stage, titles and abstracts were screened against the setting and concept gates and each record was provisionally sorted as an include, a maybe or an exclude. At the second stage, full texts were assessed strictly against both gates and every provisional “maybe” was resolved to a final decision. Records that could not be retrieved despite targeted sourcing were documented as not obtainable rather than silently dropped. The full flow of records, with reasons for exclusion at the full-text stage, is shown in Figure 1.

Titles, abstracts and full texts were screened independently by two reviewers (A.M. and M.A.) applying the strict two-gate rule. Disagreements were resolved by discussion until consensus was reached and any remaining borderline cases were adjudicated against the full text. Because the boundary between assisted living and internationally labelled residential care is not standardised, these consensus decisions are made auditable rather than hidden: every borderline residential-care report is listed explicitly in the comparison-context sources (Section 3.5) rather than being quietly excluded.

### 2.5. Data Charting and Synthesis

Data were charted into a structured form capturing the identifier, title, year, country, study design, the medication-safety concepts addressed and the specific findings reported. Charting was iterative: the form was refined as the evidence was read and the categories were adjusted to fit what the studies actually reported. Because a scoping review maps rather than pools evidence, no formal risk-of-bias appraisal or meta-analysis was undertaken. Synthesis then proceeded as a step distinct from charting. The charted entries were read in full and coded by medication-safety concept, and codes that recurred across sources were grouped into higher-order categories. Through this inductive process the sources were classified into five thematic domains, which structure the corresponding subheadings of the Results (Section 3.4.1, Section 3.4.2, Section 3.4.3, Section 3.4.4 and Section 3.4.5): the burden of polypharmacy and inappropriate prescribing; threats to medication safety; intervention studies; antimicrobial stewardship and medication-process safety; and care coordination and regulation. Within each domain the findings were summarised narratively and cross-tabulated by study design to produce the evidence-gap map. These five themes were not pre-specified; they emerged during the iterative charting process as recurring foci became apparent, and the two reviewers (A.M. and M.A.) discussed and agreed each thematic assignment, resolving the small number of borderline cases by consensus. The review was supervised by M.W. and V.F. Throughout the manuscript, sources are cited by sequential reference numbers in MDPI style, numbered in order of first appearance in the text. The 22 included assisted-living sources and the 11 comparison-context sources are kept distinguishable through separate tables in Section 3.2 and Section 3.5 rather than through their reference numbers, while the numbering runs continuously through a single reference list.

## 3. Results

### 3.1. Selection of Sources

The two database searches returned 1789 records (PubMed 773, Web of Science 1016), and the curated reference collection contributed a further 37 full texts. After 789 duplicates were removed from the database stream and 20 from the supplementary stream, 1000 records went forward to title and abstract screening. Fifty-three were excluded at that stage, leaving 947 marked for full-text assessment. Of the full texts already to hand, 450 were assessed strictly against both gates: 13 met the narrow assisted-living definition, 11 were set in residential care, nursing homes or long-term care and were retained as comparison context, and 426 were excluded (setting or concept outside scope, reviews or protocols). A further 62 titles were requested for retrieval; 28 were obtained from the search list and three through hand-searching, while 31 could not be obtained. Of the 31 retrieved full texts, 20 were excluded with reasons, namely 13 because the setting was not assisted living in the narrow sense (nursing home, residential aged-care facility, long-term care, hospital or emergency department), four because they were secondary literature, two because medication was not a central concept, and one because it was an opinion piece without data, while 11 were included at full text screening. Two of those eleven duplicated sources already included, leaving nine genuinely new sources. The strict review therefore rests on 22 included assisted-living sources: 13 already available plus nine newly retrieved (eight from the retrieval list and one from hand-searching, the Shed-MEDS pilot), with 11 residential-care sources carried separately as comparison context. Figure 1 presents the full PRISMA-ScR flow and read from top to bottom it traces how the initial records were reduced step by step, through duplicate removal, title and abstract screening and full-text assessment, down to the final included set, while also recording the reasons for exclusion at the full-text stage.

### 3.2. Characteristics of Included Sources

The 22 assisted-living sources span 2011 to 2026 and come from four countries, with the United States and Finland dominating the evidence base. Designs are mixed and mostly observational, reflecting the state of a young field: cross-sectional studies are the most common, supported by a small cluster of randomised trials, several qualitative studies and one systematic review with meta-analysis, alongside usability, feasibility, pilot, cost-effectiveness, protocol and health-service-typology studies. Table 1 summarises these characteristics, giving a one-glance profile of the evidence base by publication period, country of origin, predominant design and thematic focus. Table 2 then lists every included source individually with its identifier, short title, year, country, design and synthesis theme, so that every claim made in the text can be traced back to the study behind it.

### 3.3. Distribution of the Evidence: A Gap Map

Cross-tabulating the 22 sources by theme and study design exposes a pattern that the theme counts alone conceal (Table 3). The point of the table is to show where the evidence concentrates and where it is missing, so its empty cells carry as much meaning as its filled ones. Three features stand out. First, the evidence is overwhelmingly observational: cross-sectional and observational designs account for nine of the twenty-two sources, and once the single systematic review, the lone usability study and the protocol are set aside, experimental evidence is confined to a small number of trials and pilots. Second, and more striking, all five randomised controlled trials originate in Finland [9,11,15,23,24] and derive from a small cluster of educational-intervention studies conducted, in substance, by a single research group. The randomised evidence in the strict assisted-living literature therefore comes substantially from one research programme, which materially narrows the experimental base. Third, the United States, which contributes 13 of the 22 sources and so more than half the field, has produced no randomised trial in this setting at all. Its interventional contribution amounts to two pilot studies, the 2014 antibiotic pre–post study [13] and the 2026 Shed-MEDS deprescribing pilot [16], while its remaining 11 sources are observational, qualitative or regulatory. Deprescribing as an intervention in assisted living in the narrow sense is, on this evidence, represented by a single 2026 pilot. Read correctly, the map shows how the evidence is distributed, not how strong it is: because no risk-of-bias appraisal was undertaken, it counts studies rather than weighting them, and the patterns it reveals concern the location and design of the evidence rather than its internal validity.

The gap map makes the empty cells as informative as the full ones. There is no randomised or experimental evidence for the safety theme (PRN prescribing, administration usability, compliance aids) and none for antimicrobial stewardship in assisted living specifically, and these are the two areas where the process-of-care risk is arguably most acute. Temporally the field is young and accelerating, since 11 of the 22 sources, or half of them, appeared from 2021–2026, against seven from 2011–2015 and four from 2016–2020, so the setting is only now beginning to attract sustained attention. Geographically it is narrow: the United States and Finland together supply 19 of 22 sources (86 per cent), with only Canada (2) and the United Kingdom (1) otherwise represented and no strict assisted-living evidence at all from continental Europe, Asia or Australasia, since those regions appear only in the comparison-context set (Section 3.5).

### 3.4. Thematic Synthesis

Reading across the 22 sources, five themes recur. They are not watertight compartments, and several studies could sit under two headings, but they capture where the research energy has gone and, just as tellingly, where it has not.

#### 3.4.1. The Burden of Polypharmacy and Inappropriate Prescribing

A first cluster of studies establishes that the problem is real and sizeable in assisted living. Cross-sectional work in the United States documents high medication counts and a substantial prevalence of polypharmacy, together with non-trivial rates of falls and hospitalisation [2]. Finnish data show that potentially severe drug–drug interactions are common among residents and cluster with the number and type of medications prescribed [8]. The same Finnish programme framed the burden of inappropriate, anticholinergic and multiple psychotropic drug use clearly enough to justify a dedicated trial [11]. A recurring observation is that psychotropic prescribing in assisted living tracks the prescribing culture of the surrounding care system, and local nursing-home patterns in particular, rather than the needs of the individual resident [10], which already hints at the coordination problem that later themes make explicit.

#### 3.4.2. Threats to Medication Safety

A second cluster turns from how much is prescribed to how safely it is managed. Antipsychotic use in residents with dementia is a persistent concern, and a synthesis of the evidence underlines both its prevalence and the difficulty of curbing it without structural support such as staff training and clearer accountability [21]. Pro re nata, or “as needed”, psychotropic prescribing is widespread, and a seven-state United States study found that a substantial share of residents had a PRN psychotropic order, and although most orders carried a written indication, a meaningful minority did not, leaving considerable discretion in the hands of front-line staff [12]. The mechanics of administration matter too. A usability study of an electronic medication-delivery unit found that cognitive impairment sharply reduced residents’ ability to operate it successfully, a reminder that technological fixes assume a capacity many residents do not have [20]. Qualitative work with older people in Scottish very sheltered housing similarly shows that multi-compartment compliance aids are experienced in complicated ways and that high-quality evidence for their benefit on adherence or clinical outcomes is thin [22]. Across these studies the theme is consistent: safety in assisted living depends heavily on people and processes that are neither clinically trained nor closely supervised.

#### 3.4.3. Intervention Studies

The third cluster is among the most encouraging and, at the same time, the thinnest. The strongest signal comes from a Finnish nurse-education trial, which reported that training care staff to recognise and reduce harmful medications lowered harmful medication use and was associated with a reduction in falls [9]; its feasibility and baseline work confirmed that such a programme can be delivered in real assisted-living facilities with a predominantly cognitively impaired population [15]. The most recent addition is a United States pilot of the Shed-MEDS deprescribing intervention in residents with dementia, in which a nurse-practitioner-led review of a heavily medicated group generated deprescribing recommendations that were accepted at high rates by both surrogates and prescribers, with quality-of-life signals moving in the expected direction [16]. Cross-national comparison work on antipsychotic use in assisted living versus long-term care rounds out the picture by showing how resident and facility characteristics shape inappropriate prescribing [4]. Taken together, these studies suggest that structured education and pharmacist- or nurse-led review can move the needle, yet they are few, mostly small and concentrated in a couple of national contexts, which is exactly the kind of gap a scoping review is meant to surface.

#### 3.4.4. Antimicrobial Stewardship and Medication-Process Safety

The fourth cluster concerns antibiotics and the medication-handling process around them. An early United States study of four assisted-living communities mapped the incidence of antibiotic prescribing and the clear potential for improvement, with a preliminary quality-improvement component [13]. More recent process work has begun to unpick the antibiotic prescribing and administration pathway in assisted living step by step, identifying the many discrete points at which oversight could be strengthened [14]. The broader safety of the medication-handling process is captured by a foundational study of medication administration errors in assisted living, which tied error rates directly to the scope of staff training [6]. Set against these process concerns, nationally representative data show that older adults in assisted living use and spend more on medications than their community-dwelling counterparts, underscoring how much medication this lightly staffed setting is actually handling [3]. That antibiotics and administration errors form a distinct cluster is itself informative: they are tractable, measurable targets, which may be precisely why the research has concentrated here.

#### 3.4.5. Care Coordination and Regulation

The final and largest cluster returns to the structural argument that opened this review. Several studies show that how assisted living is regulated and staffed shapes medication outcomes directly. A national typology of health-service regulation in assisted living documents just how heterogeneous the rules are across jurisdictions, spanning medication administration, medication review and licensed-nurse staffing, which makes it hard even to compare facilities, let alone hold them to a common standard [1]. An analysis of state regulation links a more consistent regulatory presence to fewer potentially burdensome transitions for residents at the end of life [5], while a Canadian survey of medical-care-provider involvement in Ontario assisted-living homes shows how variable the clinical point of contact is and how that variability bears on medication review and hospitalisation [7]. On the intervention side, a Finnish educational programme was evaluated both for its effect on psychotropic use and related costs [24] and for cost-effectiveness, with honest reporting that reductions in inappropriate use did not translate cleanly into quality-adjusted life-year gains [23]. A United States study protocol to develop a toolkit for engaging residents and families in medication safety extends the coordination theme into applied practice [25]. The consistent message is that medication safety in assisted living is not, at root, a pharmacological puzzle but an organisational one: the drugs can be reviewed and reduced, but only if someone with the right training and remit is clearly responsible for doing so.

#### 3.4.6. How the Themes Fit Together

Read in sequence, the five themes are not a list but a single causal chain, and it is worth making that connection explicit rather than leaving it for the reader to infer. The burden studies define the input: heavy and frequently inappropriate medication loads, driven partly by the prescribing culture of the surrounding care system rather than by individual need [2,8,10,11]. The safety studies show that the machinery meant to manage that input is fragile, because administration and monitoring devolve to staff who are neither clinically trained nor closely supervised and because the technological aids intended to compensate assume capacities many residents lack [12,20,21,22]. The intervention studies then expose the common active ingredient in everything that works: each successful approach operates by inserting a trained reviewer, whether a nurse, a nurse practitioner or a pharmacist, into a system that otherwise has none [4,9,15,16]. The stewardship and process studies localise the same deficit at the most measurable point of care, antibiotic decisions and administration errors, which is plausibly why research has concentrated there rather than because they are the most consequential harms [3,6,13,14]. And the coordination and regulation studies close the loop by showing that these outcomes track, above all else, whether a jurisdiction requires such review and staffing in the first place [1,5,7,23,24,25]. The themes therefore converge on one conclusion rather than five: the binding constraint on medication safety in assisted living is structural, not pharmacological, which is precisely the take-home message set out in the Introduction. Every subsequent argument in the Discussion is a consequence of this single observation.

### 3.5. Comparison-Context Sources (Not Counted as Includes)

Eleven reports were clearly about medication safety, polypharmacy, deprescribing or antimicrobial stewardship but were set in residential care, nursing homes or long-term care rather than assisted living in the narrow sense. Under the strict setting gate these are not assisted-living includes and are excluded from all counts and from the thematic synthesis above. They are nonetheless listed transparently, in Table 4, for two reasons: they document the borderline decisions that the two-reviewer consensus flagged at the assisted-living/residential-care boundary, and they provide a comparison backdrop against which the sparseness of the strict assisted-living evidence becomes clear. Several of them speak directly to questions the assisted-living field has barely begun to answer, and that in itself is part of what this review finds: potentially inappropriate prescribing in care homes [26], antimicrobial stewardship in long-term care [27,28,29], deprescribing feasibility [30] and medication review in nursing homes [31,32].

## 4. Discussion

### 4.1. Principal Findings

This review set out to map the extent, nature and distribution of research on medication safety in assisted living in the narrow sense and to establish where the evidence clusters and where it thins. In brief, it found a small, geographically concentrated and largely observational evidence base whose central weakness is structural rather than pharmacological. Mapped strictly to its own setting, the assisted-living medication-safety literature is real but modest, uneven and revealing. Twenty-two sources, from only four countries, establish beyond much doubt that residents carry heavy and often inappropriate medication loads, that the machinery for managing those medications safely is fragile, and that the few interventions tested so far, among them nurse and staff education, pharmacist- and nurse-led review and structured deprescribing, can help. What the field largely lacks is scale. The intervention evidence rests on a handful of small trials clustered in Finland and the United States, the deprescribing evidence in this precise setting amounts to little more than a recent pilot, and much of the process-safety work concentrates on antibiotics and administration errors, arguably because they are the most measurable targets rather than the most important ones.

The gap map (Table 3) sharpens this into a concrete methodological finding with direct bearing on external validity. Every randomised trial in the strict assisted-living literature comes from Finland, and from a small cluster of educational-intervention studies at that; the United States, despite supplying more than half the sources, has generated no randomised trial in this setting and only two pilots. Two implications follow. First, the single most influential piece of causal evidence, namely that staff and nurse education can reduce harmful medication use, has essentially been tested in one country and under one regulatory and staffing model, so its transportability to the very different, lightly regulated United States assisted-living environment is genuinely uncertain rather than assumed. Second, deprescribing, the intervention most directly implied by the burden data, has been trialled in this exact setting only once, as a 2026 pilot. A field cannot be said to have an evidence base for an intervention on the strength of a single pilot and naming that plainly is part of what a scoping review is for.

The thread running through every theme is coordination. Burden accumulates because prescribing is fragmented across off-site physicians with no single clinician responsible for reviewing the whole regimen; safety is threatened because administration falls to lightly trained staff; interventions succeed precisely when they insert a trained reviewer, be it a nurse, a nurse practitioner or a pharmacist, into a system that otherwise has none; and the coordination and regulation studies show that outcomes track the presence or absence of exactly that structure. Assisted living has, in effect, absorbed the clinical complexity of a nursing home while keeping the oversight architecture of housing. This answers the organising question posed in the Introduction, now on the basis of the evidence rather than as an assertion; although stated up front for clarity, the argument emerged inductively from the charting and synthesis rather than being imposed as an a priori framework: the binding constraint is a missing structure of clinical accountability, not a missing drug, and every implication that follows flows from it.

### 4.2. The Regulatory Gap as a Public-Health Consideration

This is where the public-health framing earns its place, though it should be stated as a reasoned interpretation rather than a demonstrated causal claim. In most nursing homes, mandated registered-nurse coverage and required periodic drug-regimen review provide a floor of medication oversight. Assisted living, regulated at the state or regional level and often without any registered-nurse requirement, has no comparable floor and the typology and regulation studies in this review show just how much the rules vary. The plausible consequence is a systematic, population-level exposure to preventable medication harm that is patterned not by clinical need but by regulatory geography: two residents with identical medication lists and identical frailty may receive very different levels of oversight depending only on which jurisdiction and which facility they happen to live in. The studies assembled here are consistent with that reading and make it reasonable, but they are mostly observational and cannot on their own prove that regulatory variation causes differential harm. What can be said with more confidence is that the most promising levers appear to be organisational and regulatory at least as much as pharmacological and they point towards minimum standards for medication review, a defined clinical point of accountability and staffing rules matched to the acuity actually present. This structural argument also sits within a broader public-health logic. Populations are ageing across high-income countries, and the accompanying rise in chronic disease and functional dependency is well documented [37,38]. Because these demographic shifts are largely foreseeable, the demand they place on supportive housing and long-term care can in principle be anticipated and the oversight structure strengthened incrementally rather than reactively [39]. At the same time, the COVID-19 pandemic showed how an unpredictable epidemiological shock can rapidly expose the fragility of congregate settings and force urgent action, with assisted-living communities facing particular regulatory and operational strain [40]. Medication oversight is one of the structural dimensions that both routine demographic planning and emergency preparedness should explicitly encompass.

### 4.3. Comparison with Nursing Homes and the European Context

The decision to define assisted living strictly is what makes this comparison legible. These findings can also be set against the broader review literature: systematic reviews of deprescribing in older people with frailty and of prescribing optimisation in care homes report that structured, pharmacist- or nurse-led medication review can reduce inappropriate prescribing, although effects on hard clinical outcomes remain uncertain [17,18]. Our setting-specific map is consistent with that picture but adds a qualification, since the interventions those reviews synthesise were tested overwhelmingly in nursing homes and general care-home or community populations, not in assisted living, so their transportability to a setting without mandated on-site nursing cannot be assumed. The present review therefore does not duplicate the existing evidence but marks its boundary. A great deal of the surrounding evidence sits in “care homes”, “residential aged care” or “long-term care”, where the assisted-living stratum is invisible or merged with nursing-home populations that carry mandated oversight. The 11 comparison-context sources in Section 3.5 illustrate the point directly: they cover potentially inappropriate prescribing, antimicrobial stewardship, deprescribing and medication review in residential and long-term care, and they are, in several cases, more developed than anything yet done in assisted living itself. Folding that literature into the counts would have produced a reassuringly large map and a misleading one, because it would have credited assisted living with an evidence base and an oversight structure that belong to a different setting. Holding the line at assisted living in the narrow sense yields a smaller map that tells the truth about the setting: that it has been studied far less than its risk profile warrants and that much of what looks like relevant evidence is actually about somewhere else.

The geographic skew of the map deserves interpretation rather than mere description, because part of it is an artefact of language rather than of care. “Assisted living” is largely a North American regulatory category, and most European systems deliver broadly comparable services under different names and different rules, from sheltered and extra-care housing in the United Kingdom, to “betreutes Wohnen” in Germany, to various forms of service or sheltered housing across the Nordic countries. A direct estimate of how many European older people live in assisted living specifically is not available, precisely because assisted living is not a distinct statistical category in most European systems and is generally subsumed within long-term care. What the European data make clear is instead the scale of the surrounding demand: driven by population ageing, public spending on long-term care across the EU is projected to rise from 1.7% of GDP in 2022 to 2.6% by 2070 [41]. These are not simply translations of the same thing: they differ in how nursing input, medication administration and clinical oversight are organised and financed, which is precisely why most European reports in this field fell into the comparison-context set rather than the strict core. Even the Finnish studies that supply six of the twenty-two included sources describe a service-housing model (“palveluasuminen”) that maps only approximately onto the American term, so the apparent national diversity of the evidence is narrower than it first appears, and in effect just two systems, the United States and Finland, carry the field. The implication is easy to miss and, on our reading, important: the thinness of strict assisted-living evidence outside North America is partly a terminological artefact, not necessarily a sign that European systems neglect the problem, since comparable populations are being studied, only under categories a strict “assisted-living” lens does not capture. This reading cuts two ways. It cautions against treating the map as evidence that Europe ignores medication safety in supported older-adult housing; but it also suggests that the specific structural gap this review identifies, that of a high-acuity population housed in a lightly regulated, non-nursing setting, is likely to be most sharply expressed in the United States, where the “assisted living” category is at once the largest and the least uniformly regulated. Because no European-language search was undertaken and these analogous settings were deliberately held out of the core, this comparison is offered as structural context, not as a systematic finding.

### 4.4. Strengths and Limitations

The main strengths of this review are its discipline about setting, resting on a transparent, consistently applied two-gate rule screened independently by two reviewers, with every borderline residential-care report listed explicitly rather than quietly dropped, together with its explicit mapping of where the evidence concentrates and where it is absent. The limitations are stated plainly. First, the search was limited to two databases (PubMed and Web of Science); the omission of Embase and CINAHL in particular may affect completeness, particularly of nursing-focused literature, and a wider multi-database search might surface additional regional, nursing or grey studies. The retrieved evidence converged strongly on a few countries and designs, but that convergence cannot itself rule out a search artefact. Because 19 of the 22 sources come from only two countries, the United States and Finland, the findings should be transferred to health systems with different organisational and regulatory structures only with caution. Second, retrieval was incomplete: 31 potentially eligible full texts could not be obtained, so a small number of eligible studies may be missing from the map. Third, consistent with scoping-review methodology, no formal risk-of-bias appraisal was performed, so the review describes the shape of the evidence without judging the internal validity of individual studies; the gap map in Table 3 therefore counts studies without weighting them by quality. Finally, the strict definition, while a strength for fidelity, necessarily narrows the evidence base and should not be read as implying that residential-care findings are irrelevant to assisted living, only that they are not, strictly, about it.

### 4.5. Implications for Research and Practice

For research, the gaps almost name the studies needed to fill them: adequately powered deprescribing and coordination trials designed specifically for assisted living rather than borrowed from nursing homes; pragmatic evaluations of who is best placed to hold medication-review accountability in a setting without on-site nursing, whether a pharmacist, a nurse practitioner or a visiting physician; cross-jurisdictional cohort studies that link registered-nurse and medication-review requirements to adverse-drug-event rates, which would move the regulatory argument from plausible interpretation to tested hypothesis; and work outside the United States and Finland, which between them currently carry the large majority of the evidence. From a public-health standpoint the most urgent of these are the cross-jurisdictional studies linking registered-nurse and medication-review requirements to adverse-drug-event rates, because they would test the regulatory hypothesis that is this review’s central concern, followed closely by adequately powered deprescribing trials designed for assisted living rather than borrowed from nursing homes. For practice and policy, the review points towards defining a clear clinical owner for periodic medication review, embedding structured deprescribing and antimicrobial-stewardship pathways suited to a lightly staffed setting and, at the regulatory level, setting medication-oversight expectations that reflect the acuity assisted living now routinely carries.

## 5. Conclusions

Assisted living has increasingly come to house a high-acuity medication population without acquiring the oversight infrastructure to match. This scoping review, held deliberately to the narrow definition of the setting, maps a body of evidence that is genuine but sparse: 22 sources that confirm a heavy and often inappropriate medication burden, expose the fragility of the systems meant to manage it, and show that the interventions most likely to help are precisely those that insert trained clinical review and coordination into a setting that otherwise lacks them. The most consequential gap is not a missing drug or a missing guideline but a missing structure of accountability and it appears to be patterned by regulation as much as by clinical practice. Reducing polypharmacy-related harm in assisted living will therefore require rigorous, setting-specific deprescribing and coordination trials on the research side and, on the policy side, a public-health commitment to close the gap between the clinical complexity residents bring and the oversight the setting is currently required to provide.

## Figures and Tables

**Table 1:** Summary characteristics of the 22 included assisted-living sources.

Included assisted-living sources	22 (13 already available at full text plus 9 newly retrieved: 8 from the search list and 1 by hand-searching)
Publication period	2011–2026
Country of origin	United States (13), Finland (6), Canada (2) and the United Kingdom (1)
Predominant designs	Cross-sectional studies, randomised controlled trials, qualitative studies and a systematic review/meta-analysis, plus usability, feasibility, pilot, cost-effectiveness, protocol and health-service-typology studies
Thematic distribution	Coordination and regulation (6), then burden, safety, interventions and stewardship (4 each)
Comparison-context sources	11 residential-care, nursing-home or long-term-care reports, carried separately and not counted as assisted-living includes

**Table 2:** Included assisted-living sources, grouped by synthesis theme. Full references are listed at the end of the manuscript.

Reference Number	Source (Title)	Year	Country	Design	Theme
[1]	A National Typology of Health Service Regulation in Assisted Living	2024	USA	Qualitative	Coordination
[2]	Polypharmacy in assisted living and impact on clinical outcomes	2018	USA	Cross-sectional	Burden
[3]	Medication Costs and Use of Older Americans in Assisted Living Settings: a Nationally Representative Cross-Sectional Study	2023	USA	Cross-sectional	Stewardship
[4]	Prevalence of, and Resident and Facility Characteristics Associated With Antipsychotic Use in Assisted Living vs. Long-Term Care Facilities: A Cross-Sectional Analysis from Alberta, Canada	2017	Canada	Cross-sectional	Interventions
[5]	State regulations and assisted living residents’ potentially burdensome transitions at the end of life	2023	USA	Cross-sectional	Coordination
[6]	Medication administration errors in assisted living: scope, characteristics, and the importance of staff training	2011	USA	Observational (medication-error study)	Stewardship
[7]	Medical Care Provider Involvement in Ontario Assisted Living Homes: A Descriptive Cross-Sectional Survey Analysis	2024	Canada	Cross-sectional	Coordination
[8]	Potentially severe drug-drug interactions among older people and associations in assisted living facilities in Finland: a cross-sectional study	2016	Finland	Cross-sectional	Burden
[9]	Nurse education to reduce harmful medication use in assisted living facilities: a randomised controlled trial on falls and cognition	2015	Finland	RCT	Interventions
[10]	To What Extent Do Local Nursing Home Prescribing Patterns Relate to Psychotropic Prescribing in Assisted Living?	2021	USA	Cross-sectional	Burden
[11]	Reducing inappropriate, anticholinergic and psychotropic drugs among older residents in assisted living facilities: study protocol for a randomized controlled trial	2012	Finland	RCT	Burden
[12]	Pro re nata (PRN) prescribing and administration of psychotropic medications in assisted living: a seven-state study	2022	USA	Cross-sectional	Safety
[13]	Antibiotic prescribing in 4 assisted-living communities: incidence and potential for improvement	2014	USA	Pre-post/Pilot	Stewardship
[14]	Understanding and mapping the antibiotic prescribing and administration process in assisted living facilities	2025	USA	Qualitative (process mapping)	Stewardship
[15]	Feasibility and baseline findings of an educational intervention to optimise drug treatment among residents in assisted living facilities	2014	Finland	RCT (baseline/feasibility)	Interventions
[16]	Pilot implementation of the Shed-MEDS deprescribing intervention for persons with dementia in assisted living facilities	2026	USA	Pilot intervention	Interventions
[20]	Assessing the impact of cognitive impairment on the usability of an electronic medication delivery unit in an assisted living population	2014	USA	Usability study	Safety
[21]	Addressing Antipsychotic Use in Assisted Living Residents with Dementia	2015	USA	Systematic review/Meta-analysis	Safety
[22]	The experiences and beliefs of older people in Scottish very sheltered housing about using multi-compartment compliance aids	2018	UK	Qualitative	Safety
[23]	Cost-effectiveness of an educational intervention to reduce potentially inappropriate medication	2022	Finland	RCT	Coordination
[24]	The effect of educational intervention on use of psychotropics in defined daily doses and related costs—a randomized controlled trial	2022	Finland	RCT	Coordination
[25]	Developing a toolkit to improve resident and family engagement in the safety of assisted living: Engage (study protocol)	2022	USA	Study protocol	Coordination

(Burden = burden of polypharmacy and inappropriate prescribing; Safety = threats to medication safety; Interventions = intervention studies; Stewardship = antimicrobial stewardship and medication-process safety; Coordination = care coordination and regulation). Full references are listed at the end of the manuscript.

**Table 3:** Evidence-gap map: distribution of the 22 included assisted-living sources by synthesis theme and study design.

Theme	Cross-Sec./Obs.	RCT	Qualitative	Pilot/Feas.	Other *
Burden	3	1	—	—	—
Safety	1	—	1	—	2
Interventions	1	2	—	1	—
Stewardship	2	—	1	1	—
Coordination	2	2	1	—	1
Total	9	5	3	2	3

* Other = systematic review/meta-analysis, usability study and study protocol. — indicates that no study was identified in that theme–design combination.

**Table 4:** Comparison-context sources: residential-care, nursing-home or long-term-care reports retained for context and not counted as assisted-living includes.

Reference Number	Source (Title)	Year	Setting (Not Assisted Living i.n.s.)
[26]	Potentially inappropriate prescribing in older people with dementia in care homes: a retrospective analysis	2012	Residential aged care (RACF), Australia
[27]	Reducing inappropriate antibiotic prescribing in the residential care setting: current perspectives	2014	Residential aged care (RACF), Australia
[28]	Factors influencing antibiotic prescribing in long-term care facilities: a qualitative in-depth study	2014	Long-term care/nursing homes, The Netherlands
[29]	Are antimicrobial stewardship interventions effective and safe in long-term care facilities? A systematic review and meta-analysis	2021	Long-term care facilities (systematic review)
[30]	Deprescribing anticholinergic and sedative medicines: protocol for a Feasibility Trial (DE-FEAT-polypharmacy) in residential aged care facilities	2017	Older people (feasibility protocol), New Zealand
[31]	The Cost of Potentially Inappropriate Medications in Nursing Homes in West Occitanie	2020	Nursing homes, France
[32]	Impact of medication reviews on drug-related problems (DRPs) in older patients living in nursing homes in West Occitania	2023	Nursing homes, France
[33]	Reducing risks associated with medicines and lifestyle in a residential care population with intellectual disabilities evaluation of a pharmacy review initiative in England	2021	Care homes (intellectual disability), UK
[34]	Excessive polypharmacy and potentially inappropriate prescribing in 147 care homes: a cross-sectional study	2021	Care homes, Scotland
[35]	Analysis of medication management system data to determine potentially inappropriate medication use and hospitalization among older adults living in residential care homes for the elderly population	2025	Residential care homes for the elderly (RCHEs), Hong Kong
[36]	Quality of prescribing and health-related quality of life in older adults: a narrative review with a special focus on patients with atrial fibrillation and multimorbidity	2025	Residential aged care/mixed (systematic review), Ireland

## Data Availability

No new data were created or analyzed in this study. Data sharing is not applicable to this article.

## Supplementary Materials

The following supporting information can be downloaded at: https://www.mdpi.com/article/10.3390/geriatrics11040109/s1, Table S1. Full electronic search strategies for PubMed/MEDLINE and the Web of Science Core Collection.

## Abbreviations

The following abbreviations are used in this manuscript:

JBI	Joanna Briggs Institute
LTC	Long-Term Care
MeSH	Medical Subject Headings
OSF	Open Science Framework
PCC	Population, Concept, Context
PIM	Potentially Inappropriate Medication
PRISMA-ScR	Preferred Reporting Items for Systematic Reviews and Meta-Analyses extension for Scoping Reviews
PRN	Pro Re Nata (as required)
RCT	Randomised Controlled Trial

## Appendix A

**Section**	**#**	**PRISMA-ScR Checklist Item**	**Reported in (Section)**
**Title**	**1**	Identify the report as a scoping review.	Title page; article type “Review”; Abstract and Section 2.1 state that a scoping review was conducted.
**Abstract**	**2**	Provide a structured summary that includes (as applicable): background, objectives, eligibility criteria, sources of evidence, charting methods, results and conclusions that relate to the review questions and objectives.	Abstract (Background, Objective, Methods, Results, Conclusions).
**Introduction**	**3**	Describe the rationale for the review in the context of what is already known. Explain why the review questions/objectives lend themselves to a scoping review approach.	Section 1 (Introduction).
**Introduction**	**4**	Provide an explicit statement of the questions and objectives being addressed with reference to their key elements (e.g., population or participants, concepts and context) or other relevant key elements used to conceptualise the review questions and/or objectives.	Section 1 (3 review questions); PCC elements in Section 2.2.
**Methods**	**5**	Indicate whether a review protocol exists; state if and where it can be accessed (e.g., a Web address); and if available, provide registration information, including the registration number.	Section 2.1 (protocol and registration statement, incl. OSF registration).
**Methods**	**6**	Specify characteristics of the sources of evidence used as eligibility criteria (e.g., years considered, language and publication status) and provide a rationale.	Section 2.2 (PCC, two-gate rule); Section 2.3 (2010–2025 window).
**Methods**	**7**	Describe all information sources in the search (e.g., databases with dates of coverage and contact with authors to identify additional sources) as well as the date the most recent search was executed.	Section 2.3 (PubMed, Web of Science Core Collection; search date 26 May 2026; hand-searching).
**Methods**	**8**	Present the full electronic search strategy for at least 1 database, including any limits used, such that it could be repeated.	Section 2.3 (full PubMed and Web of Science strings).
**Methods**	**9**	State the process for selecting sources of evidence (i.e., screening and eligibility) included in the scoping review.	Section 2.4; Figure 1 (PRISMA-ScR flow).
**Methods**	**10**	Describe the methods of charting data from the included sources of evidence (e.g., calibrated forms or forms that have been tested by the team before their use, and whether data charting was done independently or in duplicate) and any processes for obtaining and confirming data from investigators.	Section 2.5 (structured charting form, iterative refinement).
**Methods**	**11**	List and define all variables for which data were sought and any assumptions and simplifications made.	Section 2.5 (charting fields: identifier, title, year, country, design, concepts, findings).
**Methods**	**12**	If done, provide a rationale for conducting a critical appraisal of included sources of evidence; describe the methods used and how this information was used in any data synthesis (if appropriate). [OPTIONAL]	Not applicable; no critical appraisal was undertaken, consistent with scoping-review methodology (Section 2.5).
**Methods**	**13**	Describe the methods of handling and summarising the data that were charted.	Section 2.5 (inductive thematic grouping into 5 themes).
**Results**	**14**	Give numbers of sources of evidence screened, assessed for eligibility and included in the review, with reasons for exclusions at each stage, ideally using a flow diagram.	Section 3.1; Figure 1 (PRISMA-ScR flow diagram).
**Results**	**15**	For each source of evidence, present characteristics for which data were charted and provide the citations.	Section 3.2; Table 1 (aggregate characteristics); Table 2 (per-source characteristics).
**Results**	**16**	If done, present data on critical appraisal of included sources of evidence (see item 12). [OPTIONAL]	Not applicable; no critical appraisal was undertaken (see item 12).
**Results**	**17**	For each included source of evidence, present the relevant data that were charted that relate to the review questions and objectives.	Section 3.2, Section 3.3 and Section 3.4; Table 2; Table 4 (comparison context).
**Results**	**18**	Summarise and/or present the charting results as they relate to the review questions and objectives.	Section 3.3 and Section 3.4 (themes and evidence gap map); Table 3.
**Discussion**	**19**	Summarise the main results (including an overview of concepts, themes and types of evidence available), link to the review questions and objectives and consider the relevance to key groups.	Section 4 (Discussion).
**Discussion**	**20**	Discuss the limitations of the scoping review process.	Section 4 (Limitations subsection).
**Discussion**	**21**	Provide a general interpretation of the results with respect to the review questions and objectives, as well as potential implications and/or next steps.	Section 5 (Conclusions).
**Funding**	**22**	Describe sources of funding for the included sources of evidence, as well as sources of funding for the scoping review. Describe the role of the funders of the scoping review.	Funding statement (end matter).
