# Supplementary material for: Fragmented Care, Fragmented Safety: Care Coordination and Deprescribing Strategies to Reduce Polypharmacy-Related Harm in Assisted Living—A Public Health Perspective
Source: Geriatrics (Basel). 2026 Aug 18;11(4):109. doi: 10.3390/geriatrics11040109 (PMC13511910; doi:10.3390/geriatrics11040109)
Supplement: Supplementary file 1 [file geriatrics-11-00109-s001.zip › geriatrics-4468527-supplementary.pdf]

Supplementary Material Table S1

Fragmented Care, Fragmented Safety: Care Coordination and Deprescribing Strategies to Reduce Polypharmacy-Related Harm in Assisted Living. A Public Health Perspective

Table S1. Full electronic search strategies for PubMed/MEDLINE and the Web of Science Core Collection. Both strings were run on 26 May 2026 with the publication window restricted to 2010–2025. The setting block was deliberately kept broad (taking in residential-care and care-home terms alongside assisted living) to maximise sensitivity at the search stage; the narrow assisted-living definition was then applied at screening rather than in the search string itself.

Database	Full search string (as executed, 26 May 2026; window 2010–2025)
**PubMed (MEDLINE)**	("Assisted Living Facilities"[Mesh] OR "assisted living"[tiab] OR "residential care"[tiab] OR "residential aged care"[tiab] OR "care home*"[tiab] OR "care-home*"[tiab] OR "sheltered housing"[tiab] OR "Homes for the Aged"[Mesh]) AND ("Polypharmacy"[Mesh] OR polypharmac*[tiab] OR "Deprescriptions"[Mesh] OR deprescrib*[tiab] OR "Potentially Inappropriate Medication List"[Mesh] OR "potentially inappropriate medication*"[tiab] OR "inappropriate prescribing"[tiab] OR "Medication Errors"[Mesh] OR "medication safety"[tiab] OR "adverse drug event*"[tiab] OR "adverse drug reaction*"[tiab] OR "drug regimen review*"[tiab] OR "medication review*"[tiab]) AND ("2010/01/01"[PDAT] : "2025/12/31"[PDAT])
**Web of Science Core Collection (TS = Topic)**	TS=("assisted living" OR "residential care" OR "residential aged care" OR "care home*" OR "sheltered housing" OR "homes for the aged" OR "aged care facilit*") AND TS=(polypharmac* OR deprescrib* OR "potentially inappropriate medication*" OR "inappropriate prescribing" OR "medication safety" OR "medication error*" OR "adverse drug event*" OR "adverse drug reaction*" OR "drug regimen review*" OR "medication review*") AND PY=(2010-2025)

In addition to the two database searches, a curated reference collection assembled during protocol development and targeted hand-searching of reference lists were used to identify further eligible reports. Because these supplementary sources were not bound by the database date filter, they could post-date the 2010–2025 window.
